# Protein Markers for Insulin-Producing Beta Cells with Higher Glucose Sensitivity

**DOI:** 10.1371/journal.pone.0014214

**Published:** 2010-12-06

**Authors:** Geert A. Martens, Lei Jiang, Katrijn Verhaeghen, Joanne B. Connolly, Scott G. Geromanos, Geert Stangé, Laurence Van Oudenhove, Bart Devreese, Karine H. Hellemans, Zhidong Ling, Christiaan Van Schravendijk, Daniel G. Pipeleers, Johannes P. C. Vissers, Frans K. Gorus

**Affiliations:** 1 Diabetes Research Center, Brussels Free University (VUB), Brussels, Belgium; 2 University Hospital Brussels (UZ Brussel), Brussels, Belgium; 3 Waters Corporation, Manchester, United Kingdom; 4 Laboratory for Protein Biochemistry and Biomolecular Engineering (L-ProBE), Universiteit Gent, Gent, Belgium; University of Bremen, Germany

## Abstract

**Background and Methodology:**

Pancreatic beta cells show intercellular differences in their metabolic glucose sensitivity and associated activation of insulin production. To identify protein markers for these variations in functional glucose sensitivity, rat beta cell subpopulations were flow-sorted for their level of glucose-induced NAD(P)H and their proteomes were quantified by label-free data independent alternate scanning LC-MS. Beta cell-selective proteins were also identified through comparison with rat brain and liver tissue and with purified islet alpha cells, after geometrical normalization using 6 stably expressed reference proteins.

**Principal Findings:**

All tissues combined, 943 proteins were reliably quantified. In beta cells, 93 out of 467 quantifiable proteins were uniquely detected in this cell type; several other proteins presented a high molar abundance in beta cells. The proteome of the beta cell subpopulation with high metabolic and biosynthetic responsiveness to 7.5 mM glucose was characterized by *(i)* an on average 50% higher expression of protein biosynthesis regulators such as 40S and 60S ribosomal constituents, NADPH-dependent protein folding factors and translation elongation factors; *(ii)* 50% higher levels of enzymes involved in glycolysis and in the cytosolic arm of the malate/aspartate-NADH-shuttle. No differences were noticed in mitochondrial enzymes of the Krebs cycle, beta-oxidation or respiratory chain.

**Conclusions:**

Quantification of subtle variations in the proteome using alternate scanning LC-MS shows that beta cell metabolic glucose responsiveness is mostly associated with higher levels of glycolytic but not of mitochondrial enzymes.

## Introduction

Insulin-producing beta cells are the body's central glucose sensors. Key to their glucose sensing, is their dependence on low-affinity glucose phosphorylation by glucokinase [1], [2]. Freshly isolated beta cells reveal heterogeneity in metabolic glucose sensitivity [3]. Intercellular variations in glucokinase abundance and activity give rise to proportionate variations in concentration-response curves for glucose-induced NAD(P)H, insulin synthesis and secretion [2], [4]–[7]. This functional heterogeneity is subject to regulation in vivo – as shown in animals exposed to sulfonylurea [8]-, and likely contributes to normal glucose tolerance. Beta cells with higher glucose sensitivity are also less susceptible to oxidative damage [6], [9]. Glucose intolerance and type 2 diabetes is thought to involve progressive beta cell exhaustion, provoked by sustained metabolic overload, leading to loss of glucose sensitivity [10]. Understanding how intrinsic glucose sensitivity is reflected in the beta cell proteome can guide us to markers to study functional adaptations in the beta cell mass in vivo, and ultimately to therapy that can regulate it. Such natural variations in glucose sensitivity are likely explained by minor variations along a normal distribution of enzyme abundances rather than by dichotomous absence/presence of key enzymes. Disclosing such specializations requires precise and accurate quantifications. Data-independent, alternate-scanning LC-MS is an ion current based mass spectrometric analysis method that offers label-free quantification of molar protein abundances [11], [12]. The present study first evaluated if alternate scanning LC-MS achieves sufficient accuracy and precision to measure functional sub-specializations within a pure cell type at the protein level. A second aim was to describe protein markers that are quantitatively associated with the beta cells' metabolic responsiveness to glucose. Finally, through a tissue-comparative analysis, an attempt was made to catalog protein markers with selective expression in insulin-producing beta cells.

## Results

### Dynamic range of detected proteomes

**Figure 1:**
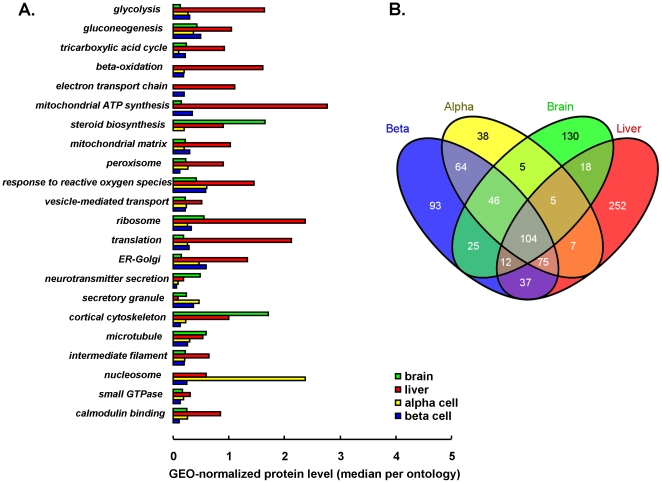
Overview of detected proteomes. Panel **A**. shows functional pathways that are statistically enriched in the detected proteomes (p<0.001); for each pathway (gene ontology), the average protein level, after geometric normalization, is shown for each of 4 tissues so that relative importance of these pathways can be quantitatively estimated between tissues. Venn diagram in panel **B**. shows the number of protein identifications in each tissue and their overlaps denoting overall proteome similarity.

Molar protein amounts in FACS-purified rat islet beta and alpha cells were measured, and compared to whole liver and brain proteomes [11]. A total of 943 proteins were identified in liver, brain, alpha and beta cells. The number of identifications in a single tissue ranged from 346 (brain) to 527 (liver). Within each tissue, the most abundant proteins were 200 to 600-fold more present than the lowest detectable ones that still could be reliably quantified (Table 1). The important overlap of the 4 tissue proteomes reflects that identified proteins typically belong to abundant functional pathways, with in decreasing order: cytoskeleton constituents, metabolic enzymes and proteins involved in protein biosynthesis and intracellular signaling (Fig. 1a). Islet endocrine alpha and beta cells showed highest similarity (Fig. 1b). Liver had the highest number of identifications; consequently, molar amounts in liver were in average 4-fold lower than in endocrine and neural cells (Table 1). Quantitative comparison of tissue proteomes thus requires normalization to reference proteins with stable expression between tissues (Fig. S1); 6 such references belonging to 3 different functional pathways, were selected for geometric [13] normalization of molar amounts as specified in Experimental procedures and Fig. S1: 2 cytoskeleton- (*Tubb5*, *Pfn1*), 2 signaling- (*Ywhae, Rab1b*) and 2 protein synthesis (*Ppia*, *Hspa8*)-associated proteins, and spread over a concentration range of 2 decades.

**Table 1:** Overview of protein identifications and quantifications.

		α cells	β cells	liver	brain	High β	Low β
**Number of proteins**							
identified		353	467	527	346	254	256
quantified		342	434	505	341	232	229
* **Amount of protein**							
median		0.001484	0.001954	0.001521	0.001711	0.002814	0.002577
minimum		0.000057	0.000092	0.000159	0.000098	0.000069	0.000120
** maximum		0.027730	0.052581	0.025761	0.027860	0.042239	0.046572
dynamic range (log)		2.63	2.75	2.21	2.45	2.78	2.59

*target protein (ng) detected on column/total amount protein (ng) detected on column.

**excluding top 1% percentile.

A first round of LC-MS analysis compared FACS-purified pancreatic alpha and beta cells (n = 3) to whole liver and brain tissue pools. A second independent analytical round compared rat beta cells that were sorted for their metabolic responsiveness (glucose-induced NAD(P)H) shortly after their isolation. ‘High’ and ‘low beta’ indicate respectively beta cells with higher or lower metabolic glucose responsiveness, as reflected by their level of 7.5 mM glucose-stimulated NAD(P)H production (See Fig. S2, Methods). The table shows number of proteins identified (above limit of detection), quantified (above limit of quantification) and general quantitative properties of the proteomes.

### Validity of alternate-scanning LC-MS-based beta cell proteome quantification

**Figure 2:**
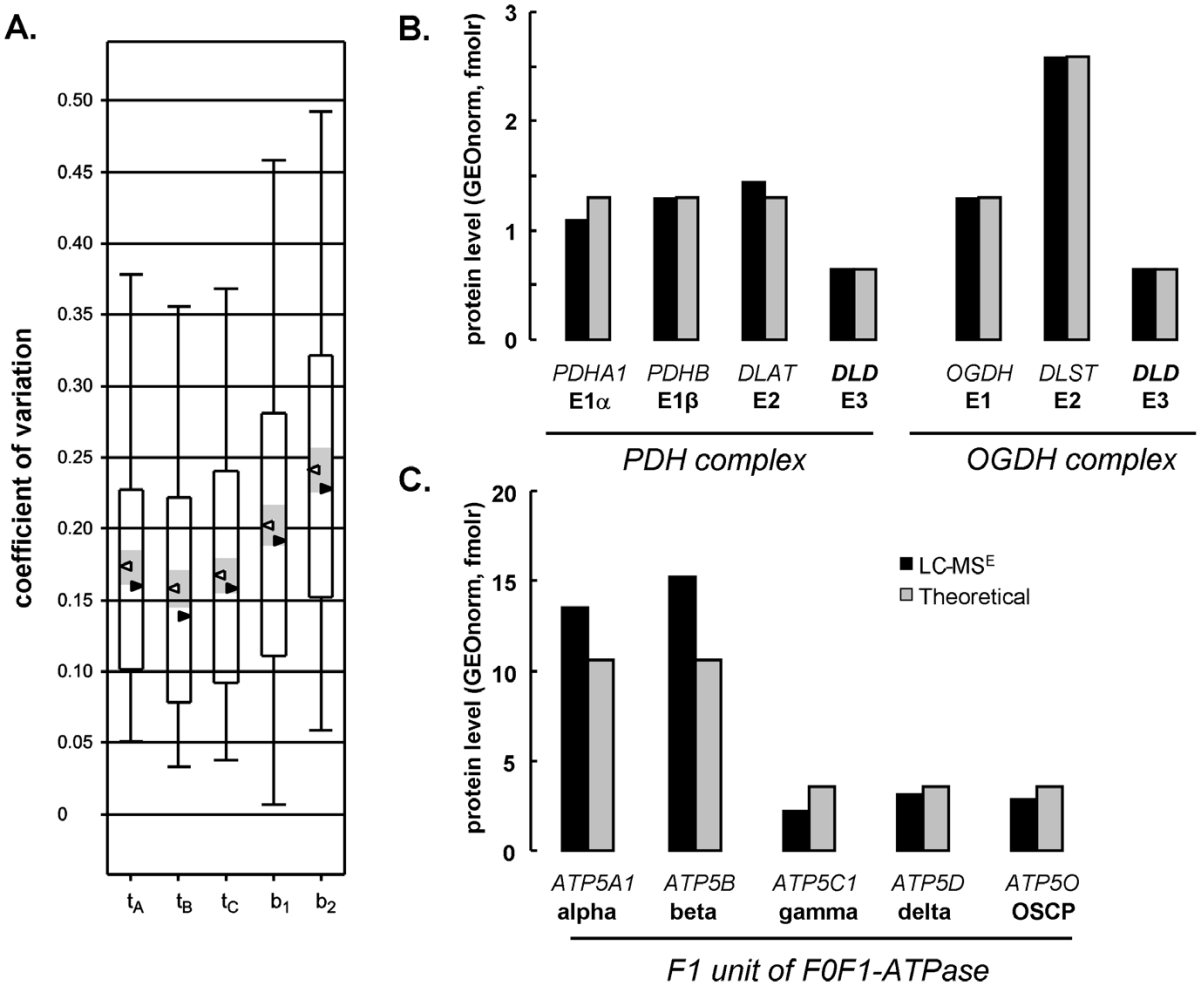
Validity of LC-MS quantifications. **A. Imprecision (error)**: beta cell proteins of 3 independent cell isolations (A, B, C) were injected in triplicate. Box and whiskers plots t_A_, t_B_ and t_C_ represent analytical variation on technical replicate LC-MS injections. Median and average values are represented by black and white triangles, respectively, with 95% confidence interval shown in grey. Total experimental imprecision (analytical + biological variation) is shown in b_1_ (normalized here for *Gapdh*) and b_2_ (total amount normalization). **B–C. Accuracy (bias)** was evaluated taking stoichiometry of multienzyme complexes as reference. Bar graphs represent geometrically-normalized protein levels (black bars, mean of n = 3) of indicated enzyme subunits measured by LC-MS in liver tissue; corresponding NCBI gene symbols are mentioned in italic capitals. Gray bars shows expected complex stoichiometry based on literature consensus. Panel **B** shows related pyruvate dehydrogenase (PDH) and oxoglutarate dehydrogenase complexes (OGDH). Panel **C** shows F1 unit of mitochondrial F0F1 ATP synthase. BCKD E2 subunit was absent in protein search library.

*Total imprecision (error)* – reflecting true biological variation plus the variation attributable to protein extraction, sample processing and LC-MS analysis – was limited to 19% (median) for the beta cell Fig. 2. *Analytical* imprecision calculated as variation solely on triplicate MS injections (Fig. 2) was limited to 14–17% (median). With such precision, molar protein expression differences between cell types down to 45% can be confidently discerned (see Experimental procedures).

Next, *accuracy (bias)* of the quantitative label-free LC-MS analyses was evaluated. The stoichiometry of subunits within multienzyme complexes such as mitochondrial F0F1-ATPase, pyruvate- (PDH) and oxoglutarate- (OGDH) dehydrogenase complexes has been extensively studied, and can serve as reference. In liver tissue, most subunits of these macrocomplexes were identified. As shown in Fig. 2b-c the relative ratios of their subunits as measured by LC-MS closely matches the previously reported stoichiometries. For the PDH complex the E1α∶E1β∶E2∶E3 ratios of 40∶40∶40∶20 were confirmed [14]. A similarly good approximation was obtained for OGDH with E1∶E2∶E3 ratios previously reported of 12∶24∶6 [15]. Also the F1-domain of the mitochondrial F0F1-ATPase, label-free quantitative LC-MS approached the known 3-fold higher expression of catalytic subunits α and β as compared to other F1-stalk subunits. Overall, accuracy for measurement of subunit stoichiometry is 104±25%.

### The proteome of beta cell subsets with higher or lower metabolic and functional glucose responsiveness

Since the applied LC-MS scanning technology can accurately detect moderate molar expression differences in a label-free, ion current fashion down to 45%, it was used to identify variations in enzyme abundances as function of the beta cells' metabolic glucose sensitivity. Glucokinase activity is to date the only in vitro [2], [4], [5] and in vivo [8] functional marker for glucose sensing capacity of individual beta cells. Beta cells were stimulated with 7.5 mM glucose – a concentration close to the Km of glucokinase - and FACS-sorted for their level of glucose-derived NAD(P)H (Fig. S2). Beta cells with higher glucose-induced NAD(P)H have a higher subcellular complexity (light side scatter) despite similar insulin stores [16]; their radius (forward light scatter) is on the average 11% larger (p<0.05) (Fig. S2a–b), indicating 1.35 times larger intracellular volume. This corresponds well with earlier studies from our group (1.25 times [2]) and others (1.32 times, [17]), and with the higher total protein level per cell (316±42 pg/cell in highly versus 193±52 pg/cell in lowly responsive beta, p = 0.12, n = 3). Even when cellular protein synthetic activities were corrected for difference in cell size, the highly glucose responsive phenotype retained a 1.5–2-fold higher insulin production per cell, reflecting size-independent intrinsic activation. For LC-MS analysis, an identical protein amount of each phenotype was injected for comparison. This resulted in equal detection of the 6 chosen reference proteins (*Ppia*, *Hspa8*, *Rab1b*, *Ywhae*, *Tubb5*, *Pfn1*): with their mean expression ratio in highly/lowly responsive cells of 1.05±0.05 (n = 3, range 0.90–1.19), the data enabled direct comparison of molar protein levels between phenotypes without additional normalization. Overall, LC-MS detected 254 (256) proteins in highly (lowly) glucose-responsive subpopulations – of which 232 (229) above limit of quantification and 90% (215) overlapping identifications (Table S2).

### Highly glucose-responsive beta cells have 50% higher expression of protein synthetic machinery

**Figure 3:**
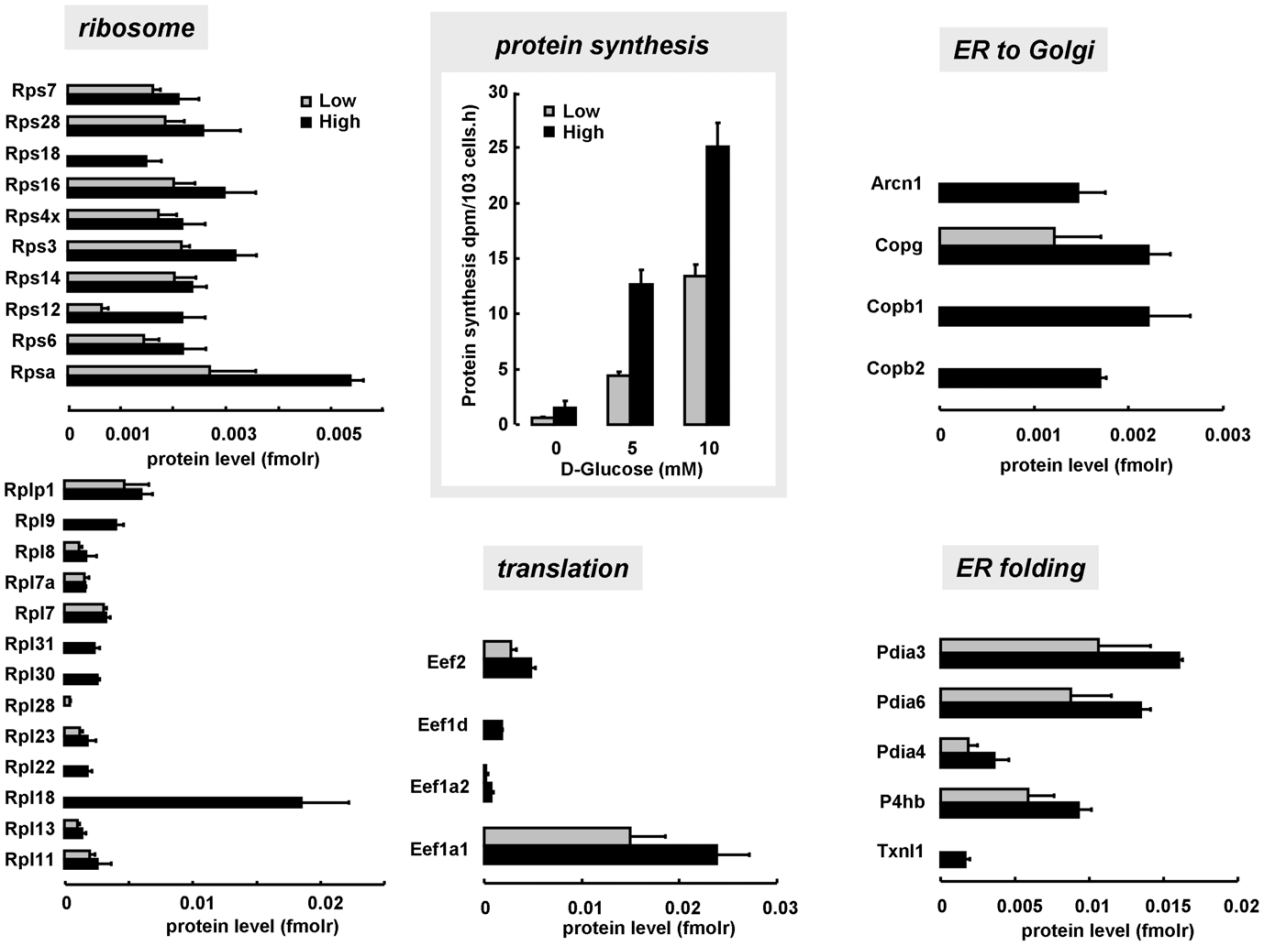
Higher protein synthetic activity of metabolically highly responsive beta cells is associated with concerted up-regulation of protein synthetic machinery. Panels from left to right counterclockwise: molar expression of small (40S) and large (60S) ribosomal subunits, mRNA translation elongation factors, endoplasmic reticulum-proteins involved in NADPH-regulated protein folding (protein disulphide isomerase family, Pdia), and subunits of the coatomer complex, involved in protein trafficking between endoplasmic reticulum and Golgi for regulated secretion. Concerted 50% protein up-regulation is associated to 2-fold higher ^3^H-Tyrosine incorporation, over 0–10 mM glucose range (blue square inset). Lowly and highly responsive beta cells represented by pale and dark blue bars respectively; absent bars indicate protein level below limit of quantification.

Highly glucose-responsive cells expressed more 40S and 60S *ribosomal protein*: 6 ribosomal subunits were only detected in highly glucose responsive beta cells; commonly detected ribosomal subunits were 1.5 times (95% CI 1.2–1.8) more abundant in highly glucose responsive beta cells. These cells also expressed 50% more translation elongation proteins (both eEF1 and eEF2) (Fig. 3). Median molar amount of 30 *endoplasmic reticulum (ER)-associated proteins* was 1.5-fold higher (average 6.9±2.4-fold, range 0.16–44 fold) in highly versus lowly responsive cells (Table S2). Examples in Fig. 3 are the NADPH-dependent protein disulphide isomerases, which mediate protein folding. Highly responsive cells also express more coatomer-complex proteins, important for normal trafficking of secretory proteins from ER to cis-Golgi [18]. In line with these changes, highly responsive beta cells synthesized 2 to 3-fold more protein than lowly responsive beta cells ((Fig. 3, Fig. S2) and [2], [16]).

### Higher glucose responsiveness correlates with higher glycolytic enzyme expression

**Figure 4:**
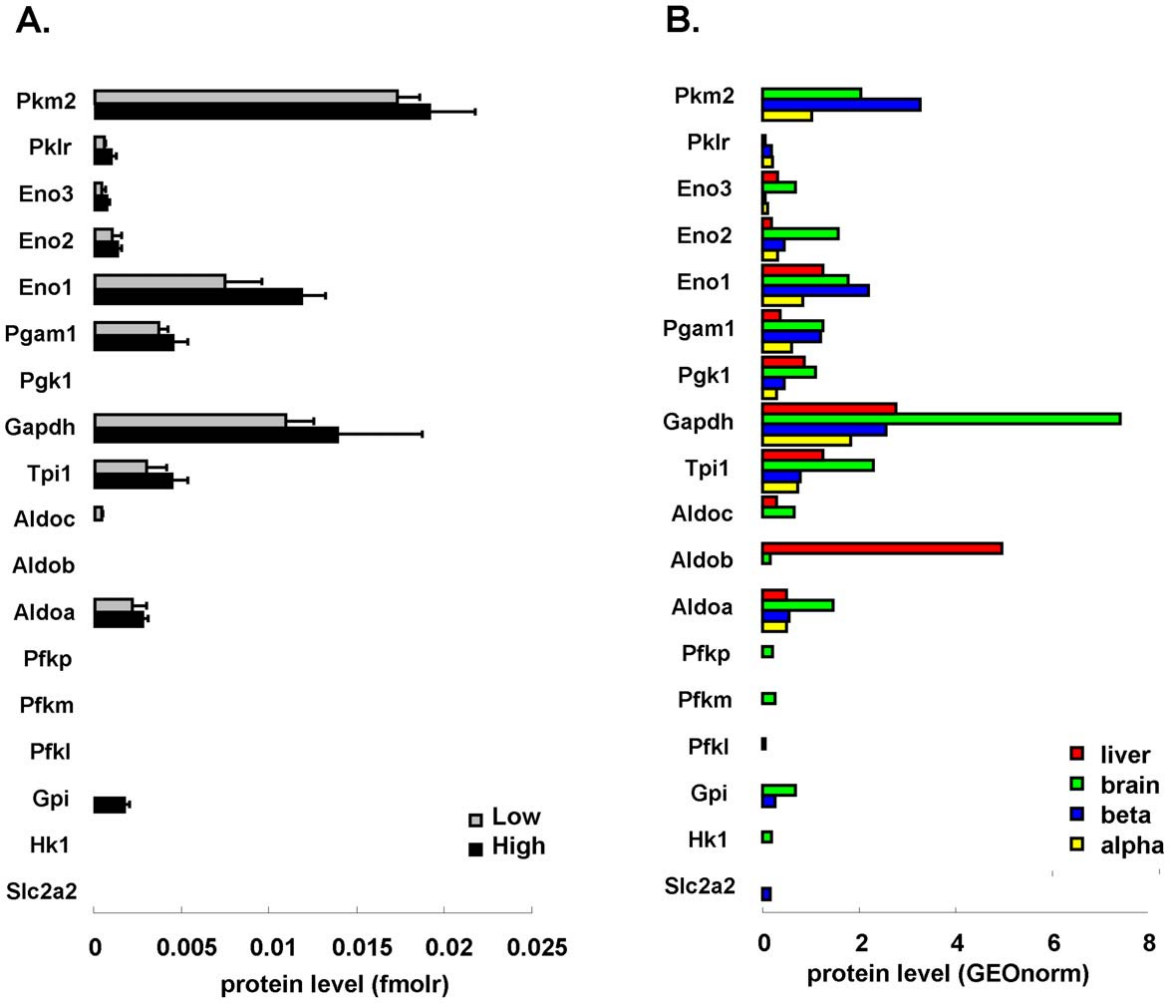
High glucose responsiveness is associated with higher molar expression of glycolytic enzymes. Comprehensive view on all enzymes of glycolysis detected in highly (black bars) and lowly (gray) glucose responsive beta cells (panel A, fmolr representing relative molar amount units, non-normalized) and in rat tissues (B, GEOnorm indicates geometrically-normalized units using 6 reference proteins). Enzymes from start to end of the pathway are represented sorted from bottom (proximal glycolysis) to top (distal glycolysis). Enzymes denoted by their NCBI gene name; absent bars indicate protein level below limit of quantification.

Glucokinase protein was below the limit of detection in liver and beta cells. The highest expression of glycolytic enzymes was found in brain. Molar levels of individual glycolytic enzymes showed strong variations with >100-fold difference in number of molecules of most abundant and lowest detectable enzymes, e.g. in liver glyceraldehyde-3-phosphate dehydrogenase (*Gapdh*) versus phosphofructokinase (*Pfkl*) (Fig. 4a–b). Relative amounts of individual enzymes were comparable in the 4 tissues studied, with overall enzymes proximal to the lytic aldolase step systematically less abundant than distal enzymes, or even undetectable – as in high/low beta cell comparison. Of note, LC-MS could correctly discern tissue-restricted isoforms, such as abundant aldolase B (*Aldob*) only in liver and distribution of enolase isoforms in brain (Fig. 4b). In pancreatic beta cells, pyruvate kinase (muscle isoform, *Pkm2*) was the most abundant glycolytic enzyme.

**Figure 5:**
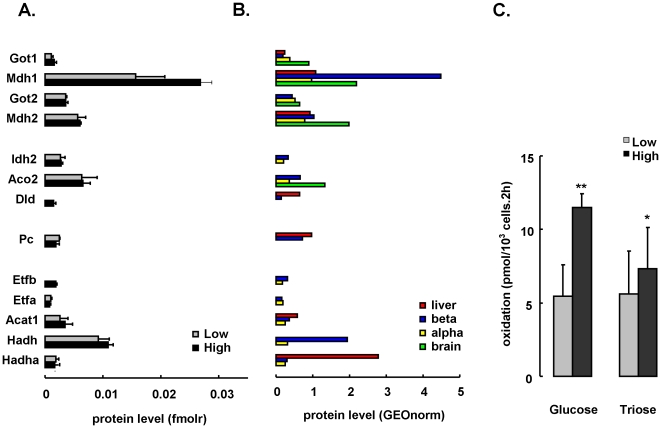
glucose responsiveness and sugar oxidation rates are not associated with mitochondrial enzyme expression. Panel A shows enzyme expression in low (gray) or high (black) responders (molar amount units), with in panel B the corresponding GEO-normalized protein levels in rat tissues. Shown are malate/aspartate shuttle enzymes (cytosolic arm:Mdh1, Got1; mitochondrial arm:Mdh2, Got2), Krebs cycle enzymes (Mdh2, Idh2, Aco2), Pdh complex unit Dld, anaplerotic enzyme pyruvate carboxylase Pc, and various beta oxidation enzymes (Hadha, Hadh, Acat1, Etfa, Etfb). Panel C shows mitochondrial oxidation rates from hexose (5 mM D-glucose) or triose (1 mM D-Glyceraldehyde) sugar in highly (black) or lowly (gray) responsive beta cells. Data represent mean±SD (n = 4) with ** p<0.001 and * p<0.05 in high versus low. Absent bars indicate protein level below limit of quantification.

Beta cells with higher metabolic glucose responsiveness expressed on average 46% (range 16–200%) higher levels of glycolytic enzymes than lowly responsive cells (Fig. 4a). Increased glycolytic enzymes were coupled to a 50% higher expression of the cytosolic arm of the malate/aspartate NADH shuttle: aspartate aminotransferase 1 (*Got1*, 44% up) and malate dehydrogenase 1 (*Mdh1*, 77% up) (Fig. 5a). High *Mdh1* appeared as metabolic specialization of beta cells with 4-fold higher expression than liver and alpha cells, and 2-fold more than brain (Fig. 5b), in line with established role of this shuttle in glucose-stimulus secretion coupling [19].

These findings suggest that glucose flux in beta cells is not only constrained at the glucokinase step, but also below. Indeed: highly glucose responsive beta cells also oxidized 30% more D-glyceraldehyde (Fig. 5c, p<0.05), a triose that enters triose phosphate isomerase step, and thus short-cuts glucokinase.

### High glucose responsiveness is not associated with higher mitochondrial enzyme expression

Despite their 2 fold higher glucose oxidation rate ([16], [20] and Fig. 5c), highly responsive beta cells show no major difference in expression of the mitochondrial branch of the NADH shuttle (*Got2* and *Mdh2*), various TCA cycle enzymes (*Mdh2, Idh2, Aco2*) or even the anaplerotic enzyme pyruvate carboxylase (*P*c) (Fig. 5a). Two flavin cofactor-containing enzymes reached detection limit only in the highly responsive cells, namely subunits from pyruvate dehydrogenase (*Dld*) and electron transfer flavoprotein (*Etfb*).

Unlike neuronal cells (Fig. 5b), beta cells express significant amounts of fatty oxidation enzymes, but their levels are not clearly associated with glucose responsiveness. LC-MS analysis confirmed the discrepantly high level of L-3-hydroxyacyl-CoA dehydrogenase (*Hadh*, formerly Hadhsc) in the beta cell beta-oxidation chain (Fig. 5b) [21]. Together with *Gapdh*, *Pkm2*, and *Mdh1*, the *Hadh* enzyme ranks among the most abundant enzymes in the beta cell.

### High glucose sensitivity of freshly isolated beta cells is in part mimicked by sustained glucose stimulation in vitro

**Figure 6:**
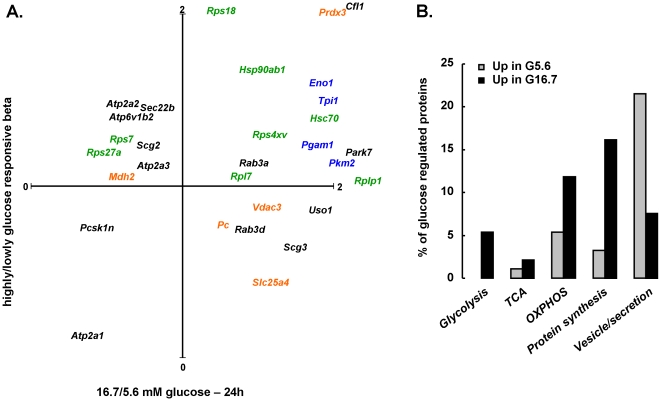
The phenotype of highly glucose responsive beta cells is partly mimicked by sustained glucose stimulation ex vivo. Of 93 proteins, 57 (61%) were up-regulated by 24 h 16.7 mM as compared to 5.6 mM glucose, and 36 (39%) down-regulated (p<0.05) in mouse islets [24]. Panel A shows their NCBI gene name in a plot, indicating their relative amount in highly/lowly glucose-responsive rat beta (Y-axis) and 16.7/5.6 mM glucose-exposed mouse islets. In blue, proteins of glycolysis; in green, protein biosynthesis; in orange, mitochondrial; in black: miscellaneous (Prdx3, Cfl1, Rps18, Rplp1, Park7 out of scale). Panel B shows % of glucose-regulated proteins (black: glucose-induced, gray: glucose-suppressed) that belong to indicated functional ontologies. For 30 of 93 proteins, molar abundances could be measured in rat highly/lowly glucose-responsive beta cells.

Beta cells that are chronically stimulated by glucose ≥10 mM become basally hyper-activated, with left-shift of the concentration-response curves for glucose-stimulated insulin synthesis, secretion and metabolic activation [6], [22], [23]. Published proteome profiles of mouse pancreatic islets, exposed for 24 h to high glucose (16.7 mM) versus basal (5.6 mM) [24], were compared to profiles of rat beta cells with high or low glucose sensitivity. Thirty proteins could be directly compared in both data sets (Fig. 6a). The major adaptations in high glucose-exposed beta cells (Fig. 6b) were increased expression of glycolysis, associated to higher expression of the protein biosynthetic machinery (ribosome, protein folding), just like in freshly isolated beta cells with higher glucose sensitivity. TCA cycle proteins were not heavily regulated.

### Quantitative view on proteins only detected in beta cells

93 of 943 proteins were below the detection limit of our LC-MS configuration in all assayed tissues, except the beta cells (Table 2). These include known beta cell markers (*Glut2, Pcksk1*, *Sytl4*) and several novel candidate beta cell markers. Functionally, these markers are enriched in ontologies involved in hormone synthesis, processing and regulated secretion. Molar abundances are generally 5 to 10-fold lower than those of reference proteins cyclophilin A (*Ppia*) and 14-3-3 protein epsilon (*Ywhae*). Yet, many fall well above our limit of quantification and even rank among the top 35% most abundant proteins of our whole data set (943 proteins): e.g. Golgi proteins such as *Copb2* and *Gosr2*, plasma membrane (associated) proteins such as *Drd3*, *Adrbk1* and *Gpsm1*, glycerol-phosphate shuttle protein *Gpd2*, and several enzymes of short chain fatty acid metabolism (*Echs1, Echdc1*).

**Table 2:** Proteins identified only in the beta cells.

*Ontology*	Symbol	Gene name	percentile	molar amount
			(%)	GEOnorm
***Endoplasmic reticulum, Golgi apparatus and secretory vesicles***				
	**Gosr2**	golgi SNAP receptor complex member 2	90	0.8865
	**Copb2**	coatomer protein complex, subunit beta 2 (beta prime)	70	0.3747
	**Ufm1**	ubiquitin-fold modifier 1	65	0.3604
	**Pcsk1**	proprotein convertase subtilisin/kexin type 1	65	0.3285
	**Sytl4**	synaptotagmin-like 4	60	0.3080
	**Sec31a**	SEC31 homolog A (S. cerevisiae)	55	0.2466
	**Sec13**	SEC13 homolog (S. cerevisiae)	55	0.2414
	**Uso1**	USO1 homolog, vesicle docking protein (yeast)	50	0.2209
	**Prrc1**	proline-rich coiled-coil 1	40	0.1830
	**Bnip1**	BCL2/adenovirus E1B 19 kDa-interacting protein 1	30	0.1268
	**Gorasp2**	golgi reassembly stacking protein 2	30	0.1327
	**Nsfl1c**	NSFL1 (p97) cofactor (p47)	20	0.0960
	**Nsfl1c**	NSFL1 (p97) cofactor (p47)	20	0.0960
	**Rnpep**	arginyl aminopeptidase (aminopeptidase B)	15	0.0768
***Intracellular signaling***				
	**Adrbk1**	Adrenergic, beta, receptor kinase 1	90	1.1449
	**Drd3**	dopamine receptor D3	90	1.2612
	**Tpt1**	tumor protein, translationally-controlled 1	80	0.5835
	**Ppp1r1a**	protein phosphatase 1, regulatory (inhibitor) subunit 1A	65	0.3683
	**Gpsm1**	G-protein signaling modulator 1 (AGS3-like, C. elegans)	55	0.2456
	**Map2k1**	mitogen activated protein kinase kinase 1	30	0.1378
	**Camk2d**	calcium/calmodulin-dependent protein kinase II delta	-	nq
	**Camk2a**	calcium/calmodulin-dependent protein kinase II alpha	-	nq
	**Camk2g**	calcium/calmodulin-dependent protein kinase II gamma	-	nq
***Metabolism***				
	**Echs1**	enoyl Coenzyme A hydratase, short chain, 1, mitochondrial	65	0.3315
	**Echdc1**	enoyl Coenzyme A hydratase domain containing 1	60	0.2786
	**Ddc**	dopa decarboxylase (aromatic L-amino acid decarboxylase)	60	0.2903
	**Gpd2**	glycerol-3-phosphate dehydrogenase 2, mitochondrial	55	0.2506
	**Aco1**	aconitase 1, soluble	50	0.2151
	**Cox5b**	cytochrome c oxidase subunit Vb	50	0.2205
	**Aldh7a1**	alpha-amino adipic semialdehyde dehydrogenase (antiquitin)	45	0.2076
	**Atp6v1e1**	ATPase, H+ transporting, lysosomal V1 subunit E1	45	0.2012
	**Slc2a2**	facilitated glucose transporter 2	35	0.1532
	**Aldh6a1**	methylmalonate semialdehyde dehydrogenase	30	0.1386
	**Ddt**	D-dopachrome tautomerase	30	0.1347
	**Acaa1a**	acetyl-Coenzyme A acyltransferase 1 (peroxisomal)	25	0.1023
	**Sardh**	sarcosine dehydrogenase	25	0.1074
	**Acads**	acyl-Coenzyme A dehydrogenase, short chain	15	0.0716
***Cell motility and cytoskeleton***				
	**Vcl**	vinculin	50	0.2149
	**Wdr1**	WD repeat domain 1	50	0.2195
	**Dctn2**	dynactin 2	45	0.2066
	**Dcx**	doublecortin	25	0.1123
	**Tpm4**	tropomyosin 4	20	0.0940
	**Fis1**	fission 1 (mitochondrial outer membrane) homolog (S. cerevisiae)	20	0.0954
	**Capzb**	capping protein (actin filament) muscle Z-line, beta	10	0.0644
	**Tpm1**	tropomyosin 1, alpha	5	0.1831
***Proteasome***				
	**Psmc3**	proteasome (prosome, macropain) 26S subunit, ATPase 3	45	0.1946
	**Psmc2**	proteasome (prosome, macropain) 26S subunit, ATPase 2	20	0.0858
	**Psmc5**	proteasome (prosome, macropain) 26S subunit, ATPase, 5	15	0.0777
***Protein synthesis - translation***				
	**Wars**	tryptophanyl-tRNA synthetase	40	0.1799
	**Gars**	glycyl-tRNA synthetase	40	0.1657
	**Tars**	threonyl-tRNA synthetase	30	0.1275
	**Asns**	asparagine synthetase	20	0.0988
	**Sars**	seryl-tRNA synthetase	15	0.0673
**6 reference proteins present in all tissues and used for GEOnormalization**				
	**Tubb5**	tubulin, b5	-	2.0417
	**Ppia**	peptidylprolyl isomerase A (cyclophilin A)	-	2.0273
	**Hspa8**	heat shock protein 8	-	1.2830
	**Ywhae**	14-3-3 protein epsilon	-	1.0002
	**Pfn1**	profilin 1	-	0.7672
	**Rab1b**	RAB1B, member RAS oncogene family	-	0.2454

93 proteins were only identified in the beta cells. Table 2 shows a selection of proteins, from statistically overrepresented (p<0.005) functional ontologies. Percentile (%) score ranks the proteins molar abundance (not normalized) to those of all other 943 identified proteins in tissue-comparative analysis with 100%  =  highest abundance. GEOnorm molar amount represents measured molar units, normalized to the 6 stably expressed proteins that are shown as reference at bottom of the table.

## Discussion

### Validity of alternate-scanning LC-MS for proteome quantification

Our study took two approaches to investigate protein markers for glucose sensitivity of beta cells: *(i)* tissue-comparative analysis to highlight (relatively) beta cell-selective expression, and *(ii)* a comparison of beta cell phenotypes that were sorted for their natural variations in metabolic glucose responsiveness. Since both comparisons rely on adequate quantification of molar protein abundances, the analytical validity of our technique was first evaluated. It provided acceptable sensitivity in terms of number of proteins identified under statistically stringent criteria. Evidently, the inherent constraints in dynamic range of mass spectrometry (MS), limit quantification of unfractionated cellular proteomes to the upper 2 to 3 orders of most abundant proteins: the dynamic ranges of 200–600 observed in our study agree with previous reports of dynamic ranges of 2.7 or 3.5 for quantification or detection respectively [25]. Of note, detection and identification of proteins also depends on extraction method and fractionation of classes of interest, which is the subject of a separate study. The MS technique was also found to be accurate - as judged from its unbiased prediction of multienzyme subunit stoichiometry - and technically precise: in combination with the standardized beta cell isolation procedures [26], overall imprecision – including true biological variation - was limited to ±20%. Consequently, differences in molar abundance of individual proteins between cells down to 45% - a value corresponding to a full width at half maximum (FWHM) of the normal distribution - can be confidently discerned.

For tissue-comparative analyses, protein abundances were geometrically normalized towards the abundance of multiple stably expressed reference proteins [13]. A pair of proteins qualifies as reference or “housekeeping” proteins when their ratio varies minimally across different cell or tissue types. Our 6 reference proteins were additionally chosen from different functional pathways to avoid selecting truly co-regulated proteins. *Ppia*, *Hspa8*, *Tubb5*, *Pfn1*, *Ywhae* and *Rab1b* were selected in the tissue-comparative data set, and successfully validated in the independently acquired data set on highly/lowly glucose responsive beta cells. Metabolic enzymes such as *Mdh2* and *Gapdh* appeared suboptimal as reference: e.g. neurons express clearly higher levels of glycolytic enzymes such as *Gapdh* (Fig. S1). Of note, if *Gapdh* were chosen as reference for highly/lowly glucose sensitive beta cells, much of their observed differences would remain unnoticed, illustrating the inevitable introduction of interpretative bias by normalizing.

### Enzymes with beta cell selective expression in terms of molar abundance

Several enzymes with established role in stimulus-secretion coupling showed beta cell-selective expression in terms of high molar abundance: L-3-hydroxyacyl-CoA dehydrogenase (short chain) (*Hadh,* formerly *Hadhsc*) [21], [27], pyruvate carboxylase (*Pc*) [28], cytosolic malate dehydrogenase (*Mdh1*) [19] and glycerol-3-phosphate dehydrogenase (*Gpd2*) [29] – the latter two central to the shuttles that transport glycolysis-derived reducing equivalents (NADH, FADH_2_) into the mitochondria. A novel and intriguing feature is the beta cell selective expression of several enzymes with presumed role in mitochondrial short-chain fatty acid metabolism: though not differentially expressed as function of natural glucose sensitivity, the roles of *Acads*, *Echs1* and *Echdc* in nutrient sensing certainly requires further study, e.g. in the context of acetoacetate-induced insulin secretion and coupling role of short-chain acyl-CoAs [30].

### Proteins associated with intercellular differences in beta cell glucose sensitivity

The classical glucose sensor enzyme, glucokinase (*Gck*) [31], was below the MS detection limit. Low molar *Gck* abundance fits with the low levels of its mRNA (3, 8 and 240 times lower than that of respectively *Gad2*, *Hadh* and *Slc2a2/Glut2*, our unpublished data). It also fits with the present finding that enzymes of proximal glycolysis are rare as compared to those downstream of the aldolase step. *Gck* is an accepted marker of beta cell glucose sensitivity [7], and its loss- or gain-of-function mutations in humans result respectively in loss of or excessive glucose sensing [31]. Our group first described the technique to flow-sort rodent beta cells for their level of metabolic glucose sensitivity: beta cells accumulate NAD(P)H when glucose stimulated, and do so in sigmoid kinetics with half-maximal NAD(P)H at glucose ∼ 7.5 mM, around the Km of *Gck* [6]. This glucose concentration-dependent activation of metabolism, results in progressive recruitment of more beta cells into a state of activated insulin synthesis [4] and secretion [5], whereby the activation threshold of individual beta cells correlates with their *Gck* expression and activity [2]. The glucose concentration-dependence of insulin production by the total beta cell population thus appears regulated by normal variations in the underlying glucose sensitivity of the individual beta cells, resulting in a response that fits the normal distribution with average activation around the Km of *Gck*. Dichotomous sorting of the beta cell population for 7.5 mM glucose-induced NAD(P)H thus yields the two tails of the normal population (Fig. S2), and provides a unique view on functionally relevant proteome variations within a pure (±95% insulin-positive) cell population.

The higher protein synthetic capacity of highly glucose sensitive beta cells was reflected in a ±50% up-regulation of all stages in their protein production line: from ribosome over translational control, to protein folding (Ppia family) in endoplasmic reticulum (ER) and ER to Golgi transport (coatomer complex) for secretion. The picture was not comprehensive - with several other likely regulators [32], such as translation initiation factors and S6 kinase not detected – but its consistency with cellular function serves as positive control.

Abundant *Mdh1* was not only characteristic for beta cells, its level also correlated with glucose sensitivity of the beta cells. In highly responsive beta cells, the 50% higher *Mdh1* was paralleled with 46% (16–200%) higher level of glycolytic enzymes. These cells oxidize glucose and D-glyceraldehyde respectively 2-fold and 30% more than lowly responsive beta cells: the former corresponds to their previously reported 2-fold higher *Gck* activity [2], the latter matches their overall higher glycolytic capacity – since D-glyceraldhyde enters at the level of glyceraldehyde-3-phosphate, independent of *Gck* [16]. Our study thus emphasizes the importance of glycolysis and transport of derived NADH into the mitochondria in regulation of glucose sensing. It is in line with the previously reported role of distal glycolytic intermediates in nutrient-regulated beta cell function [33].

These findings do not argue against the crucial role of mitochondrial nutrient oxidation and NADH (re)generation in stimulus-secretion coupling [34], but indicate that molar abundances of mitochondrial metabolic enzymes are not limiting oxidative sugar flux: several enzymes of Krebs cycle, electron transport and fatty acid oxidation could be studied, but none correlated with glucose sensitivity. Neither did the level of pyruvate carboxylase, though its anaplerotic activity is crucial for beta cell function [35].

### Cell size and intrinsic activity synergistically account for higher protein synthesis of beta cell subsets with higher glucose sensitivity

The highly glucose responsive beta cells have a larger cell volume (±30%) and contain more protein per cell than lowly responsive beta. Of note, these values are averages and appear normally distributed within each sorted subset. An identical protein amount of both phenotypes was injected into the LC-MS; the fact that this resulted in statistically similar detection of the 6 chosen reference proteins, indicates that our chosen references indeed qualify as ‘housekeepings’, and that increased size/total protein content of highly responsive beta cells is associated with a proportionate increase of all 6 ‘housekeepings’. Reference protein-normalized protein levels thus reflect the size-independent or ‘intrinsic’ functional properties. At first sight, there appeared a discrepancy between the relatively moderate (±1.5-fold) higher expression of glycolysis and protein synthetic machinery in the highly responsive beta cells, and their 2 to 3-fold higher actual protein synthetic rates (Fig. S2c). This discrepancy is resolved when also the 1.3-fold size differences are taken into account, and both factors are synergistically (1.3×1.5 = 2) combined to explain actual functional differences. This is summarized in the scheme of Fig. S2d.

### Consistency with other models

The ‘natural’ variations in glucose sensing are partly recapitulated by two in vitro models. A first is the loss of glucose sensitivity of MIN6 insulinoma cells after long-term culture [36]; the second is the proteome adaptations of primary beta cells to high glucose concentrations [24].

In the first model, Clynes et al. reported mRNA and proteome adaptations in MIN6 insulinoma cells with progressive loss of glucose sensitivity in culture [36]. Of note, they [37], and others [38], found a limited correlation between mRNA and proteome adaptations, underlining the importance of proteome studies. They identified 35 proteins as differentially regulated with varying glucose sensitivity: high sensitivity correlated with increased expression of ER proteins, including the NADPH-dependent *Pdia* family also emphasized in our study. Another parallel was that high glucose sensitivity correlated with high peroxiredoxin and superoxide dismutase 1 expression, suggesting that oxidative radical scavenging preserves normal glucose sensing. Besides, beta cells had relatively abundant expression of peroxiredoxin and superoxide dismutase enzymes as compared to other tissues (Table S1), contrary to the widely held notion of their poor oxidative scavenging capacity [39]. Also in MIN6 cells, high glucose sensitivity was associated with high *Mdh1*, elevated expression of several – but not all – glycolytic enzymes and no detectable differences in mitochondrial enzymes.

As second model, sustained stimulation with glucose >10 mM brings beta cells into a functional state that is partly reminiscent of in vivo high glucose sensitivity: left-shift of the glucose-induced metabolic activation (NAD(P)H) and insulin production [22], correlated with increased mRNA expression of glucose-catabolizing enzymes, endoplasmic reticulum (ER) proteins and secretory vesicle-associated proteins [40], [41]. A major difference is that sustained high glucose in vitro provokes basal secretory hyperactivation leading to cellular exhaustion partly due to depletion of insulin content. Waanders et al. confirmed this at the protein level [24]: 24 h high glucose (16.7 mM versus 5.6 mM) activated glycolytic enzymes, and multiple ER proteins involved in protein folding and processing; also it activated ROS-scavenging proteins such as *Prdx3* but down-regulated vesicle-associated proteins (*Vamp2*). Similarly; increased expression of glycolytic enzymes and ER proteins was found in the highly glucose sensitive beta cells, as well as increased expression of several peroxiredoxins (*Prdx3*, *Prdx5* and to a lesser extent *Prdx1* and *Prdx2*) and down-regulated level of secretory granule-associated proteins (*Vamp2*, *Chga*, *Scg3*). A major difference consists in the up-regulation of mitochondrial metabolic (TCA) enzymes after sustained high glucose, but not in the naturally highly glucose sensitive cells.

Can our findings be translated to a clinical context? Lu et al. performed a careful in-depth study of loss of beta cell glucose sensitivity in MKR mice, a rodent model of type 2 diabetes [42]. MKR beta cells initially adapt well to insulin resistance, but with time lose function leading to hyperglycemia. Their phenotype presumably reflects both the initial attempts to adapt to increased insulin demands, and with time accumulates signs of decompensation. Though comprehensive comparison is complex, MKR beta cells share global proteome fingerprints with highly glucose sensitive beta cells, with increased expression of glycolytic enzymes, *Mdh1*, many ER- and protein translation-associated proteins, and Golgi markers such as *Copb2*. A major difference here is the global suppression of TCA enzyme and electron transport proteins expression in MKR beta cells. Such loss of mitochondrial metabolic activity, in the face of up-regulated glycolysis, could explain the observed oxidative imbalance in these diabetic beta cells [6].

In conclusion: beta cells show natural variations in glucose sensitivity. The associated proteomic fingerprints can be quantitatively captured by alternate scanning LC-MS. Though the associated proteome profiles are partial - with several known regulators (e.g. glucokinase) below the MS detection limit – they are consistent with functional state. Beta cells at the upper limits of the glucose sensitivity distribution are equipped for a higher insulin synthetic capacity. They express higher levels of all glycolytic enzymes and shuttles that transport glycolysis-derived reducing equivalents into the mitochondrial. Molar abundances of mitochondrial enzymes however, are not limiting glucose oxidative flux. More generally, the present study shows that variations in glucose sensing and associated glucose-regulated functions are stably reflected in the proteome, supporting the existence of such phenotypic variations in vivo.

## Materials and Methods

### Cellular characteristics

Rats were housed according to the Belgian animal welfare regulations. Animal killing was kept to the strict minimum, after proper CO_2_-anesthesia. Use of animal cells and tissues was approved by the Commissie Proefdiergebruik (CPG) of the Vrije Universiteit Brussel (VUB), for a project entitled “in vitro and in vivo markers for beta cell death and function” (CPG approval ID 07-274-3). Rat beta and islet non-beta cells were FACS-purified as previously described [4], [5], [26]. These isolates consisted of ≥95% endocrine cells and <2% exocrine cells. Beta cell preparations consisted of 90% insulin+, 3% glucagon+, 1% somatostatin+ and 2% pancreatic polypeptide+ cells; alpha cells contained 2% insulin+, 94% glucagon+, 1% somatostatin+ and 2% pancreatic polypeptide+ cells. Functional data on ^3^H-tyrosine incorporation, D-glucose and D-glyceraldehyde oxidation rates in highly and lowly glucose-responsive beta cells were in part previously reported [16].

### Protein extraction and trypsinization

Freshly isolated rat cells were washed trice with PBS (4°C), and soluble protein was extracted in 50 µl 0.5% (w/v) RapiGest detergent in 50 mM ammonium bicarbonate (Waters Corporation, Milford, MA.) in the presence of Complete Protease Inhibitor Cocktail (F. Hoffmann–La Roche Ltd, Basel, Switserland) as specified by manufacturer's protocol and bovine DNAse II (Boehringer Ingelheim GmbH, Ingelheim am Rhein, Germany, 2 µg/mL) solution, followed by ultracentrifugation to remove cellular debris. 25 µl protein extract was reduced with 2.5 µl 100 mM dithiothreitol (DTT) at room temperature for 60 min, followed by 3 subsequent washes with 400 µl 50 mM ammonium bicarbonate and 4 µl 100 mM DTT using a 5 KDa cut-off membrane filter. This step removes protease inhibitors and most of reduced insulin molecules. Proteins were denatured by heating at 80°C for 15 min, followed by 30 min at 60°C after addition of 2.5 µl 100 mM DTT and another 30 min at ambient temperature in the dark after addition of 2.5 µl 200 mM iodoacetamide. Trypsinization was carried out overnight at 37°C (1∶25 w/w trypsin ratio) in final volume of 100 µl. Finally, RapiGest detergent was removed by acidifying digest to pH  = 2 with trifluoroacetic acid and incubation for 15 min at 37°C.

### LC-MS configuration

Nanoscale LC separation of tryptic peptides was performed with a nanoACQUITY system (Waters Corporation), equipped with a Symmetry C18 5 µm, 2 cm ×180 µm precolumn and an Atlantis C18 3 µm, 25 cm ×75 µm or a Atlantis C18 3 µm, 15 cm ×75 µm analytical reversed phase column (Waters Corporation). The samples, 2 µL full loop injection, were initially transferred with an aqueous 0.1% formic acid solution to the precolumn at a flow rate of 4 µL/min for 3 min. Mobile phase A was water with 0.1% formic acid whilst mobile phase B was 0.1% formic acid in acetonitrile. After desalting and preconcentration, the peptides were eluted from the precolumn to the analytical column and separated with a gradient of 3% to 40% mobile phase B over 90 min at a flow rate of 300 nL/min followed by a 10 min rinse with 90% of mobile phase B. The column was re-equilibrated at initial conditions for 20 min. The column temperature was maintained at 35°C. The lock mass compound, [Glu^1^]-Fibrinopeptide B, was delivered by the auxiliary pump of the LC system at 250 nL/min at a concentration of 100 fmol/µL to the reference sprayer of the NanoLockSpray source of the mass spectrometer. All samples were analyzed in triplicate.

Mass spectrometric analysis of tryptic peptides was performed using a Synapt MS mass spectrometer (Waters Corporation, Manchester, UK). For all measurements, the mass spectrometer was operated in v-mode with a typical resolution of at least 10,000 full width at half maximum. All analyses were performed in positive mode ESI. The time-of-flight analyzer of the mass spectrometer was externally calibrated with a NaI mixture from m/z 50 to 1990. The data was post-acquisition lock mass corrected using the doubly charged monoisotopic ion of [Glu^1^]-Fibrinopeptide B. The reference sprayer was sampled with a frequency of 30 s. Accurate mass precursor and fragment ion LC-MS data were collected in data independent, alternate scanning (LC-MS^E^) mode of acquisition. This method alternates the energy applied to the collision cell of the mass spectrometer between a low and elevated energy state and is described in detail elsewhere [12]. The spectral acquisition time in each mode was 0.6 s with a 0.02 s interscan delay. In low energy MS mode, data were collected at constant collision energy of 4 eV. In elevated energy MS mode, the collision energy was ramped from 15 eV to 35 eV during each 1s integration. The radio frequency amplitude applied to the quadrupole mass analyzer was adjusted such that ions from m/z 300 to 2000 were efficiently transmitted, ensuring that any ions observed in the LC-MS data less than m/z 300 were known to arise from dissociations in the collision cell.

### LC-MS data processing, protein identification and error assessment

Continuum LC-MS data were processed and searched using ProteinLynx GlobalSERVER v2.4 (Waters Corporation). Protein identifications were obtained by searching a species specific *Rattus norvegicus* databases (v15.12, 7,449 entries). Sequence information of Alcohol dehydrogenase *Saccharomyces cerevisiae* was added to the databases to afford the ability to normalize the data sets and to estimate amounts and concentration [11] and that of known contaminant proteins (serum albumin *Bos taurus* and trypsin *Sus scrofa*). A decoy was generated on the fly with every database search experiment conducted to estimated the protein false positive rate of identification [43]. Data independent scanning protein identifications were accepted when more than three fragment ions per peptide, seven fragment ions per protein and more than 2 peptides per protein were identified. Furthermore, the identification of the protein had to occur in at least 2 out of 3 replicate injections. Typical search criteria used for protein identification included automatic peptide and fragment ion tolerance settings (approximately 10 and 25 ppm, respectively), 1 allowed missed cleavage, fixed carbamidomethyl-cysteine modification and variable methionine oxidation.

Relative molar amount units are used throughout unless stated otherwise and calculated by dividing the determined molar amount for a given protein by the summed determined amount for all identified proteins as this accounts for both technical and biological variations.

*Imprecision (error)* attributable to purely analytical variation was calculated as coefficients of variation on molar values from triplicate LC-MS injections of the same sample, taken over the 10 to 90% percentile of dynamic range of quantification, i.e. excluding 10% lowest or highest abundances. Total error - including the true biological variation, as well as all sample (pre) processing procedures (islet isolation, beta cell FACS purification, protein extraction, tryptic digestion and LC-MS analysis) – was calculated on triplicate injections for all independent isolations (n = 3), after normalization. Normalization was either on total on column protein amount, with median (average) error of 22% (24%) or on that of an endogenous mid concentration range reference (*Ppia*), giving median (average) total imprecision of 19% (20%). Excluding biological variation, the sole technical LC-MS measurement impression, affords median measurement variation of 11.4%. To determine the statistically acceptable discrimination limit between two samples, a normal distribution of the proteome data was assumed. Full width at half maximum (FWHM) of these distributions was chosen as statistical discrimination limit to assess protein expression – a value corresponding to 2.35 times the median total error (19% times 2.35 = 45%).

### Selection of reference proteins and geometric normalization for tissue-comparative analysis

A first criterion to consider a pair of two proteins as reference, is that they are systematically co-regulated, i.e. that their relative expression level in different cell types remains constant. Evidently, proteins that belong to a same functional ontology are likely to be co-regulated though their absolute levels might vary between cell types; as second criterion, proteins were selected that were systematically co-regulated with proteins belonging to a different functional ontology. The relative expression ratios was calculated for all *n* proteins that were identified in all tissues (n = 104) along with their coefficient of variation between the 4 analyzed tissues for these (*n* × (*n*-1))/2 unique ratios (Fig. S1a). Protein pairs were selected with CV% <40% and extensive co-regulation with at least 7 other proteins, preferentially belonging to different functional pathways. In the data set of this study this yielded a set of ±38 possible reference proteins (Fig. S1b). Finally, 6 proteins were chosen with lowest inter-tissue CV% (ranging from 23–38%) and highest number of co-regulations, not only within their own functional pathway, but particularly with members of other functional pathways. From 3 different functional pathways, 2 proteins were chosen: cytoskeleton (*Tubb5*, *Pfn1*), cell signaling (*Ywhae, Rab1b*) and protein synthesis (*Ppia*, *Hspa8*). Each of these 6 references was then sequentially used to normalize all detected molar protein amounts (Fig. S1). Since reference proteins themselves also range in abundance over one order of magnitude, the geometric instead of the arithmetic averages of 6 normalized ratios were calculated [13].

## Supporting Information

Figure S1 Selection algorithm of reference proteins for geometric normalization in tissue-comparative analysis. A set of 104 proteins was detected in all 4 tissues. For each tissue, the relative ratio of each candidate reference was calculated with any of the other candidates. Next, average, standard deviation and associated coefficient of variation was calculated for each ratio across the 4 tissues. Couples with CV% on their ratio < 40% were marked in red, others in blue (panel A). Couples with highest number of co-regulation were subselected (panel B), including functionally co-regulated proteins e.g. of intermediary metabolism (yellow), protein synthesis (green), cytoskeleton (blue) or cell signaling (14-3-3 proteins and small GTPases). Finally, 6 reference proteins were chosen that showed the most intense co-regulation not only within their own pathway but also with members of other functional pathways.
(1.49 MB TIF)

Figure S2 Cell size-dependent and -independent functional properties of beta cell subsets with varying metabolic glucose responsiveness. Panel A shows FACS-separation of 7.5 mM glucose-responsive (high NAD(P)H)) and less responsive beta cells (low NAD(P)H). Panel B: highly responsive beta cells are on the average larger (forward light scatter, FSC, reflecting cell radius), and have higher subcellular complexity (side scatter, SSC). Both populations show similar (oxidized) riboflavin level (FAD, FMN) but show > 2-fold difference in glucose-induced NAD(P)H. Panel C: highly (purple bars) responsive beta cells produce 2 to 3 -fold more insulin than lowly responsive cells (pale blue bars), and this difference persists (1.9-fold versus lowly responsive cells, n = 3, * p < 0.05) also after correction (light purple bars) for the 1.3 ± 0.1 larger cell size of the high responders. Panel D summarizes main conclusion of this and earlier studies: highly responsive beta cell populations show intrinsic activation of glycolysis and insulin productive machinery, as reflected by ± 1.5-fold higher GEOnormalized protein levels. On top, these intrinsic phenotypic differences are further 1.3-fold amplified by the higher cell size of highly responsive cells, thus accounting the observed 2 to 3-fold higher insulin synthesis over the 0-10 mM glucose range.
(1.26 MB TIF)

Table S1 Overview of protein identifications in tissue-comparative analysis. Table shows gene symbol, protein accession and full name of all identified proteins, with the relative molar amount units in respectively alpha, beta, brain and liver. Molar amounts represent mean of 3 independent experiments, each injected in triplicate.
(0.31 MB XLS)

Table S2 Overview of protein identifications in rat beta cells, FACS-sorted for higher or lower glucose responsiveness. Table shows gene symbol, protein accession and full name of all identified proteins, with the relative molar amount units in respectively highly (HIGH) or lowly (LOW) glucose responsive beta cells. Molar amounts represent mean of 3 independent experiments, each injected in triplicate.
(0.10 MB XLS)
