# Supplementary figures and images for: Protein Markers for Insulin-Producing Beta Cells with Higher Glucose Sensitivity
Source: PLoS One. 2010 Dec 6;5(12):e14214. doi: 10.1371/journal.pone.0014214 (PMC2997773; doi:10.1371/journal.pone.0014214)

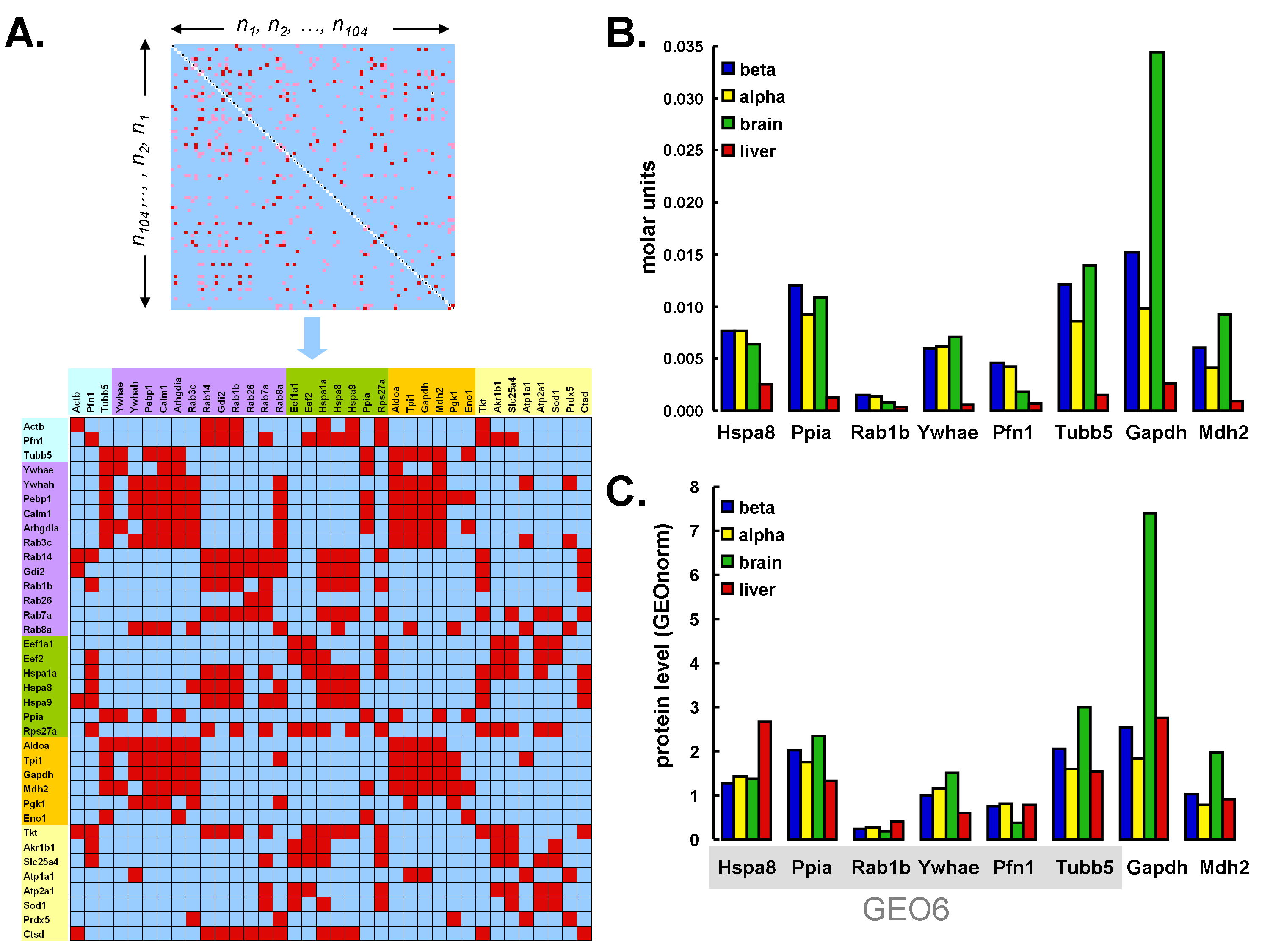

Supplement: Figure S1 — Selection algorithm of reference proteins for geometric normalization in tissue-comparative analysis. A set of 104 proteins was detected in all 4 tissues. For each tissue, the relative ratio of each candidate reference was calculated with any of the other candidates. Next, average, standard deviation and associated coefficient of variation was calculated for each ratio across the 4 tissues. Couples with CV% on their ratio < 40% were marked in red, others in blue (panel A). Couples with highest number of co-regulation were subselected (panel B), including functionally co-regulated proteins e.g. of intermediary metabolism (yellow), protein synthesis (green), cytoskeleton (blue) or cell signaling (14-3-3 proteins and small GTPases). Finally, 6 reference proteins were chosen that showed the most intense co-regulation not only within their own pathway but also with members of other functional pathways. (1.49 MB TIF) [file pone.0014214.s001.tif]

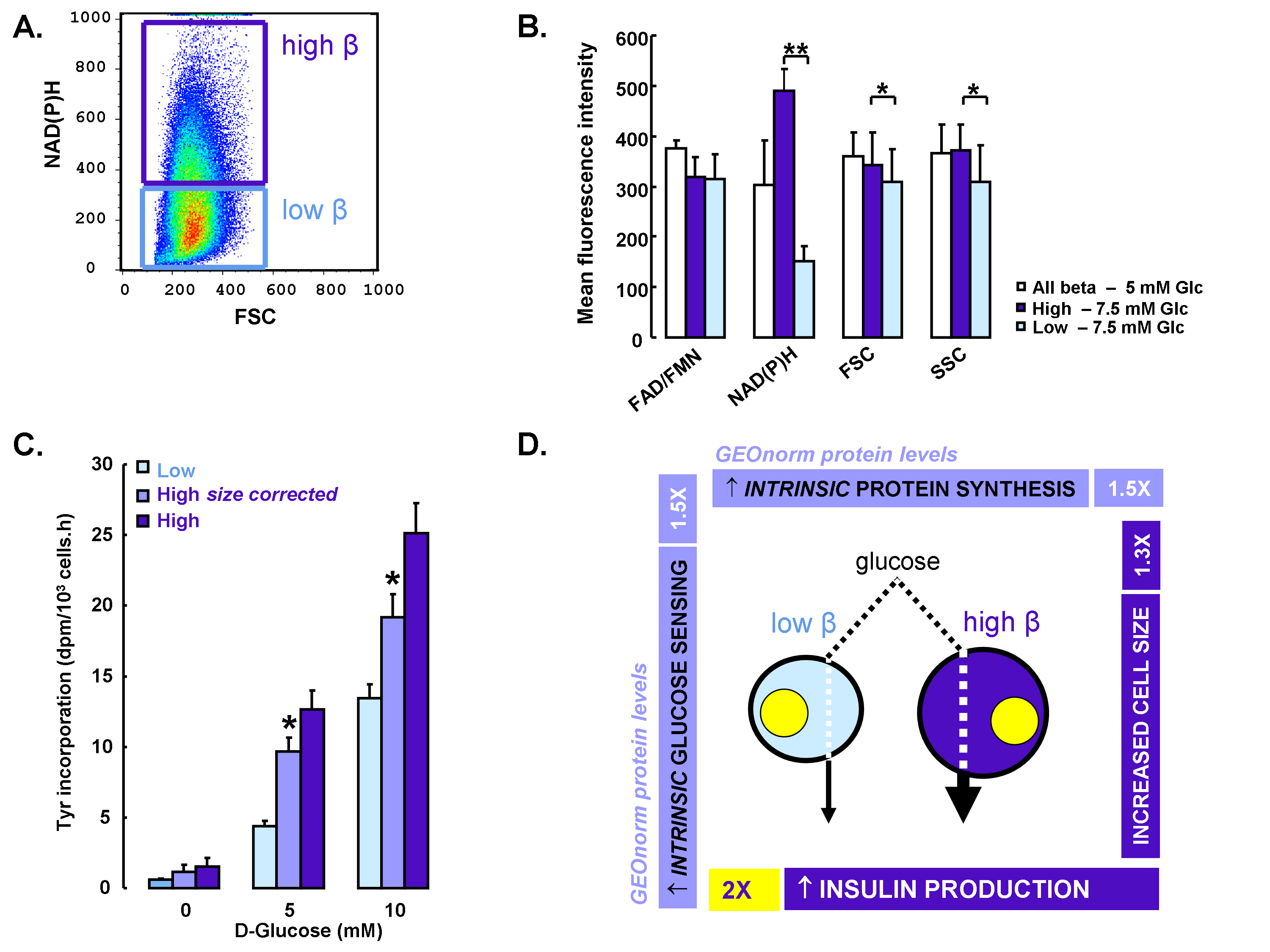

Supplement: Figure S2 — Cell size-dependent and -independent functional properties of beta cell subsets with varying metabolic glucose responsiveness. Panel A shows FACS-separation of 7.5 mM glucose-responsive (high NAD(P)H)) and less responsive beta cells (low NAD(P)H). Panel B: highly responsive beta cells are on the average larger (forward light scatter, FSC, reflecting cell radius), and have higher subcellular complexity (side scatter, SSC). Both populations show similar (oxidized) riboflavin level (FAD, FMN) but show > 2-fold difference in glucose-induced NAD(P)H. Panel C: highly (purple bars) responsive beta cells produce 2 to 3 -fold more insulin than lowly responsive cells (pale blue bars), and this difference persists (1.9-fold versus lowly responsive cells, n = 3, * p < 0.05) also after correction (light purple bars) for the 1.3 ± 0.1 larger cell size of the high responders. Panel D summarizes main conclusion of this and earlier studies: highly responsive beta cell populations show intrinsic activation of glycolysis and insulin productive machinery, as reflected by ± 1.5-fold higher GEOnormalized protein levels. On top, these intrinsic phenotypic differences are further 1.3-fold amplified by the higher cell size of highly responsive cells, thus accounting the observed 2 to 3-fold higher insulin synthesis over the 0-10 mM glucose range. (1.26 MB TIF) [file pone.0014214.s002.tif]
